# Digital solutions for tackling mental health in cancer patients and their families: The ALTHEA project

**DOI:** 10.1371/journal.pdig.0001675

**Published:** 2026-10-07

**Authors:** Ilaria Durosini, Dario Monzani, Veronica Coppini, Maria Vittoria Ferrari, Chiara Bovolenta, Asia Grigis, Giuseppe Curigliano, Paola Zagami, Elisabetta Munzone, Ida Minchella, Francesca Magnoni, Fedro Alessandro Peccatori, Isabelle Scholl, Marcel Meyerheim, Mieke Van Hemelrijck, Hannah Gsell, Lucija Gosak, Laura Ciria-Suarez, Karl Làvo, Norbert Graf, Marta Pérez-Campdepadrós, Marie Lange, Elisabeth Andritsch, Birute Brasiuniene, Andreas Charalambous, Nataliia Dovganych, Iva Kirac, Haridimos Kondylakis, Kostyantyn Kovalyov, Cristian Ochoa-Arnedo, Manolis Tsiknakis, Béata Juzyna, Ines Vaz-Luis, Philip E. V. Van Kerrebroeck, Nicole Erickson, Margarita Tareyn, Norbert Couespel, Clémentine Rialland, Luigi Grassi, Denis Horgan, Saska Benedicic Tomat, Begonya Nafria Escalera, Delia Nicoara, Claudio Lombardo, Gabriella Pravettoni, the ALTHEA Consortium

**Affiliations:** 1 Applied Research Division for Cognitive and Psychological Science, IEO, European Institute of Oncology IRCCS, Milan, Italy; 2 Department of Oncology and Hemato-Oncology, University of Milan, Milan, Italy; 3 Department of Psychology, Educational Science and Human Movement, University of Palermo, Palermo, Italy; 4 Division of New Drugs and Early Drug Development for Innovative Therapies, IEO, European Institute of Oncology, IRCCS, Milan, Italy; 5 Division of Medical Senology, IEO, European Institute of Oncology IRCCS, Milan, Italy; 6 Division of Breast Surgery, IEO, European Institute of Oncology IRCCS, Milan, Italy; 7 Gynecologic Oncology Department, IEO, European Institute of Oncology IRCCS, Milan, Italy; 8 Department of Medical Psychology, University Medical Center Hamburg-Eppendorf, Hamburg, Germany; 9 Department of Paediatric Oncology and Haematology, Faculty of Medicine, Saarland University, Homburg, Germany; 10 European Organisation for Research and Treatment of Cancer, EORTC, Brussels, Belgium; 11 Translational Oncology and Urology Research, King’s College London, London, United Kingdom; 12 Childhood Cancer International Europe, Vienna, Austria; 13 Faculty of Health Sciences, University of Maribor, Maribor, Slovenia; 14 ICOnnecta’t Digital Health Program, Catalan Institute of Oncology, Hospitalet de Llobregat, Catalonia, Spain; 15 The Bellvitge Biomedical Research Institute (IDIBELL), Psychooncology and Digital Health Group, Hospitalet del Llobregat, Catalonia, Spain; 16 Global Alliance for Mental Illness Advocacy Networks, GAMIAN-Europe, Brussels, Belgium; 17 Pediatric Cancer Centre, Sant Joan de Déu Barcelona Hospital, Barcelona, Spain; 18 Servei de Salut Mental, Sant Joan de Déu Barcelona Hospital, Barcelona, Spain; 19 Clinical Research Department, UNICANCER, Centre François Baclesse, Caen, France; 20 Equipe Labellisée Ligue Contre le Cancer, Services Unit PLATON, Cancer and Cognition Platform, Université de Caen Normandie, ANTICIPE U1086 INSERM-UCN, Centre François Baclesse, Caen, France; 21 Division of Oncology, Medical University of Graz, Graz, Austria; 22 Division of Clinical Oncology, Department of Psychiatry, Psychosomatics and Psychotherapeutic Medicine, Division of Medical Psychology, Psychosomatics and Psychotherapeutic Medicine, University Clinic of Internal Medicine, Graz, Austria; 23 National Cancer Center of Vilnius University Hospital Santaros Klinikos, Vilnius, Lithuania; 24 Faculty of Medicine, Vilnius University, Vilnius, Lithuania; 25 Department of Nursing, School of Health Sciences, Cyprus University of Technology, Limassol, Cyprus; 26 Department of Nursing, University of Turku, Turku, Finland; 27 Clinical Pharmacology & Cardio-Oncology Department, NSC “The M.D.Strazhesko Institute of Cardiology”, Kyiv, Ukraine; 28 Cardio-Oncology Center, Kyiv, Ukraine; 29 Institute for Gastroenterological Tumours (IGET), Zagreb, Croatia; 30 Department of Surgical Oncology, University Hospital for Tumors, Sestre milosrdnice University Hospital Center, Zagreb, Croatia; 31 Foundation for Research and Technology Hellas (FORTH), Institute of Computer Science, Heraklion, Greece; 32 Computer Science Department, University of Crete, Crete, Greece; 33 Blahodiyna Orhanizatsiya Blahodiynyy Fond Svit Proty Raku (WAC), Zaporizhzhia, Ukraine; 34 Charitable organization “Charitable Fund “World Against Cancer”, Zaporizhzhia, Ukraine; 35 Department of Clinical Psychology and Psychobiology, Universitat de Barcelona, Barcelona, Spain; 36 Electrical and Computer Engineering, Hellenic Mediterranean University, Heraklion, Greece; 37 Department of Research & Development, Unicancer, Paris, France; 38 Cancer Survivorship Group, INSERM U981, Gustave Roussy FR Department for the Organization of Patient Pathways, Gustave Roussy, Villejuif, France; 39 Policy Office, European Association of Urology, Arnhem, Netherlands; 40 Urology Department, University of Maastricht, Maastricht, Netherlands; 41 The European Society for Clinical Nutrition and Metabolism, ESPEN, Luxembourg, Luxembourg; 42 Association of Cancer Patients (APOZ), Sofia, Bulgaria; 43 European Cancer Organisation, Brussels, Belgium; 44 Department of Neuroscience and Rehabilitation, Institute of Psychiatry, University of Ferrara, Ferrara, Italy; 45 European Alliance for Personalised Medicine, Brussels, Belgium; 46 International Sport and Culture Association, Copenhagen, Denmark; 47 Research Department, Institut de Recerca Sant Joan de Déu, Barcelona, Spain; 48 Universitat Internacional de Catalunya, Barcelona, Spain; 49 Department of Quality Management, “Prof. Dr. Ion Chiricuta” Institute of Oncology, Cluj-Napoca, Romania; 50 Organisation of European Cancer Institutes, Brussels, Belgium; Army Medical University, CHINA

## Abstract

Despite advances in cancer treatment, the growing global cancer burden underscores the need for comprehensive and supportive care to address the needs of patients and their families. However, access to psycho-oncological support remains often limited across healthcare systems. This could be related to factors such as resource constraints and insufficient integration of psychological care into routine oncological pathways. Individual-level barriers also exist, including low awareness of available services, stigma, and heterogeneity in patients’ perceived needs and help-seeking attitudes. Nevertheless, psychological burden can significantly influence patients’ experience of the treatment pathway, influencing cognitive clarity, perceived self-efficacy, communication, risk perception, decision-making, and all areas of daily life. In this context, digital mental health solutions have emerged as promising tools to enhance accessibility, continuity of care, and personalisation of support. In this manuscript, we aim to describe the structure of the ALTHEA project (“tAckling menTal Health cancer patients and their families: digital solutions for bEtter cAre”), which focuses on the development of a novel web-based digital platform targeting cancer patients and their informal caregivers’ mental health across different stages of the care pathway. ALTHEA is distinguished by the presence of screening, classification into different support profiles, and tailored interventions within a single platform, as well as by its co-creation approach involving patients and stakeholders. In this line, the ALTHEA project represents a timely opportunity to integrate digital health solutions into psycho-oncology care, improving mental health outcomes, enhancing patient empowerment, and promoting a more holistic approach to cancer care.

## Introduction

The latest World Health Organization (WHO) statistics indicate that cancer is the second leading global cause of death [1]. Oncological diagnoses are increasing worldwide with approximately 20 million new cases diagnosed in 2022, and this number is expected to grow in the coming years [2]. In addition, pediatric cancers are on the rise, accounting for approximately 1% of global diagnoses [3]. It is estimated that 13.7 million new cases of childhood cancer will be diagnosed between 2020 and 2050 [4].

Receiving a potentially life-threatening diagnosis can lead to significant psychological and cognitive challenges. Different areas of personal life can be affected [5] and, if not properly addressed, they may negatively impact patients’ well-being, overall quality of life, and treatment adherence [6,7].

In this context, psycho-oncological interventions can support people of all ages, foster adherence to clinical recommendations, and facilitate the exploration of patients’ preferences and needs throughout the care pathways [8,9]. Nevertheless, cancer patients, survivors, family members, and informal caregivers may face barriers to accessing psychological support services at local, national, or international levels [10,11]. These challenges are often related to resource constraints and insufficient integration of psychological care into routine oncological pathways. Additionally, feelings of shame and perceived discrimination may act as further barriers, increasing the tendency toward social isolation. Low awareness of available services, stigma, and heterogeneity in patients’ perceived needs and help-seeking attitudes also contribute to these difficulties [10].

Clinical Practice Guidelines (CPGs) and recommendations support professionals in assessing and addressing psychological issues along care pathways [12]. Psychological assessment is recommended not only at the time of diagnosis, but also at different phases of the illness. Emotional and cognitive burden may change over time and may be influenced by psychological factors and patients’ age. Despite this, a critical gap remains, as most CPGs primarily focus on adult populations, with comparatively limited attention given to paediatric patients and informal caregivers.

Digital technologies have become increasingly widespread in healthcare and are increasingly being leveraged in oncology for prevention and early detection, care management, and support [13]. Digital health solutions have the potential to overcome the aforementioned barriers, but at the same time may also contribute to new forms of inequality (e.g., through varying accessibility among countries) [14].

In this context, the present manuscript describes the ALTHEA project (co-funded from the European Union’s EU4Health Programme), which aims to develop and implement a web-based digital platform to improve access to mental health screening and facilitate access to psychological and cognitive support. This platform will also be available for healthcare professionals (HCPs).

## The ALTHEA Platform

The ALTHEA platform will encompass three distinct functions.

(a) ***Psychological and cognitive assessment:*** A unique testing battery for psychological and cognitive/neuropsychological assessment of both pediatric and adult cancer patients and informal caregivers will be developed, taking into account CPGs, care guidelines, and existing validated tests and self-reported measures.
(b) ***Support profile identification:*** Users will be assigned to a support profile (0–3) based on the results of the psychological and cognitive assessments and considering complexity indicators. Complexity indicators refer to psychosocial vulnerability factors that contribute to improve the specificity of the final profile. Each support profiles corresponds to a progressively higher levels of intensity of the proposed intervention, in line with available CPGs, guidelines, and expert feedback. Four levels will be considered:
  - *Support Profile 0*: Users who will be invited to follow-up and ongoing reassessment through the ALTHEA screening module to identify early changes in psychosocial status.
  - *Support Profile 1:* Users will receive general educational materials, self-help contents and digital interventions designed to support psychological adjustment and promote adaptive coping strategies. Follow-up and ongoing reassessment will also be provided.
  - *Support Profile 2*: Users will receive self-help contents, tailored educational materials, digital interventions and guided professional support delivered through the platform by psycho-oncologists, health psychologists, and neuropsychologists. These interventions will address, for example, emotional distress, sleep quality, and cognitive functioning, and will be informed by clinical best practice.
  - *Support Profile 3*: Users will be referred to specialised care and personalised recommendations for integrated care. This approach ensures timely access to appropriate specialists for tailored support.
(c) ***Psychological support and educational material:*** The ALTHEA platform will provide tailored educational materials to patients and family members and will equip HCPs with comprehensive knowledge and necessary tools to navigate mental health issues in oncology. Guidelines, information and educational materials will inform and strengthen clinical and referral pathways, including guidance on how to refer patients to specialised psycho-oncologists and neuropsychologists.

An overview of all ALTHEA functions is provided in Fig 1.

**Fig 1:**
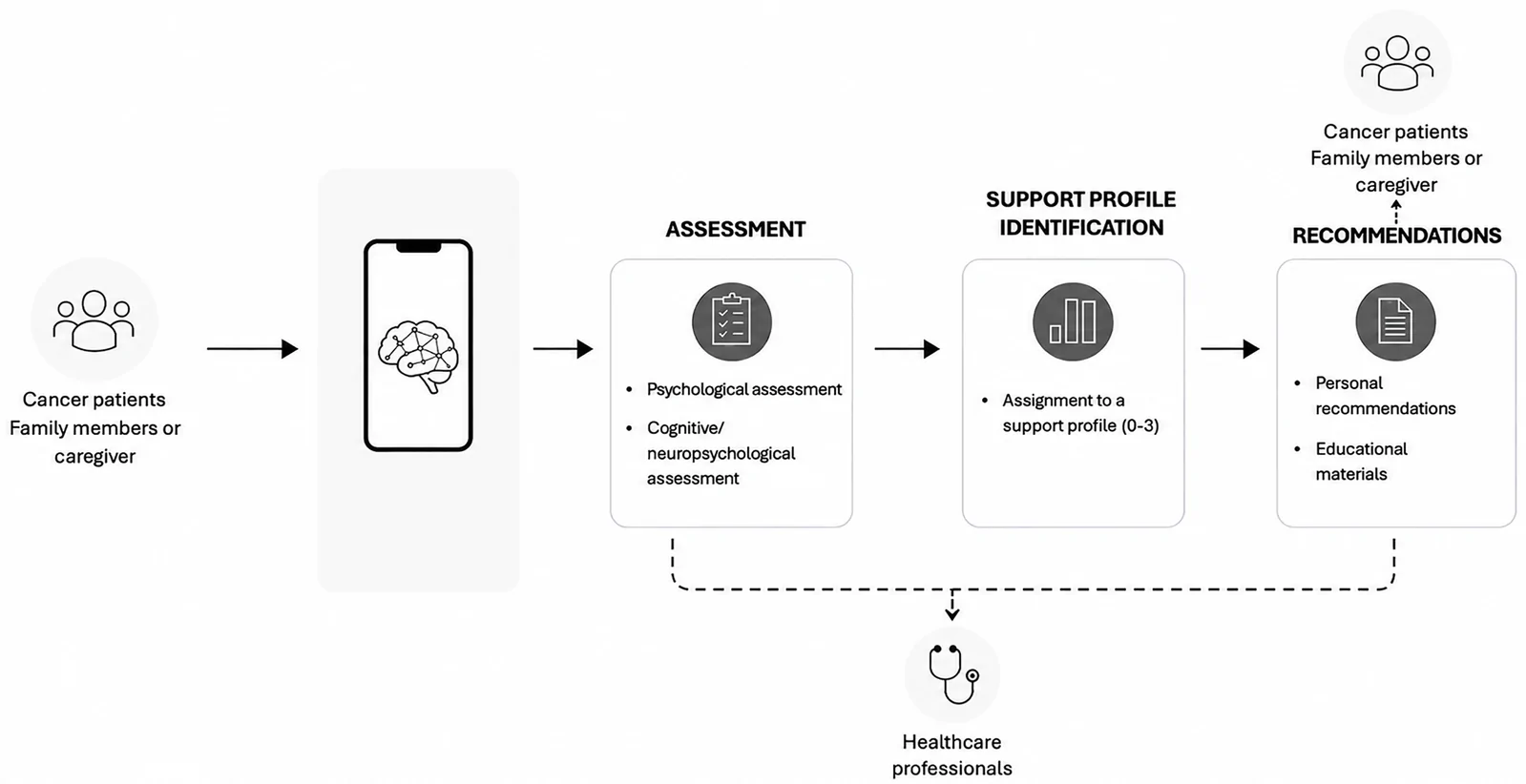
Overview of the ALTHEA digital pathways.

In order to ensure that the ALTHEA platform reflects the needs and preferences of end-users, its development will be based on the scientific literature and on data collected from patients, caregivers, and healthcare professionals. Compared to existing digital health platforms, the ALTHEA platform is distinguished by its tailored mental health screening, experience-informed support profiles identification, and specific psychological and cognitive interventions. In addition, ALTHEA targets both cancer patients and their caregivers across different stages of the care pathway, while actively involving end-users and healthcare professionals in its co-creation.

## Discussion and conclusions

The ALTHEA project is grounded in international clinical research and collaboration with health institutions, fostering the integration of research and clinical practice. Previous studies have highlighted the potential of digital platforms to improve the identification of patients’ needs and facilitate the monitoring of psychological well-being [13]. In this context, algorithm-based approaches may represent a promising strategy, although further empirical validation is needed to establish their effectiveness and generalisability [15]. Develop a digital platform for heterogeneous populations requires the inclusion of a wide range of participant groups throughout the process to ensure validity of its contents. Moreover, some people might be less inclined to be engaged due to lower levels of digital literacy [16]. These factors may contribute to digital inequalities and limit equitable access to the platform, highlighting the need for inclusive design and implementation strategies, multilingual interface, culturally adapted contents, and careful prioritisation of resources. Integrating features such as gamification, easily accessible content, and low-bandwidth solutions may further enhance the platform’s ability to deliver tailored interventions and promote sustained user engagement across diverse populations [17]. In addition, involving users with lived experience as co-creators and advisors, rather than solely as participants, can enhances the platform’s relevance, usability, acceptability, and ethical foundation.

Overall, this digital solution has the potential to contribute to more accessible and integrated psycho-oncological care by facilitating its incorporation into routine clinical pathways, supporting the early identification of psychological needs through structured screening, and improving access to tailored interventions across diverse populations. Furthermore, it may inform the development of more standardised approaches to mental health assessment in oncology and contribute to more equitable and scalable models of care. Future research will be essential to evaluate its clinical effectiveness, scalability, implementation in real-world settings, and impact on clinical outcomes.

## Supporting information

S1 Text Members of the ALTHEA Consortium. (DOCX)
