# Supplementary material for: Digital solutions for tackling mental health in cancer patients and their families: The ALTHEA project
Source: PLOS Digit Health. 2026 Oct 7;5(10):e0001675. doi: 10.1371/journal.pdig.0001675 (PMC13645018; doi:10.1371/journal.pdig.0001675)
Supplement: Supplementary file 1 [file pdig.0001675.s001.docx]

**Supporting Information**

Members of the ALTHEA Consortium

Paolo Guiddi

Florence Didier

Giorgia Mulè

Virginia Sanchini

Melissa Heifez

Johan Lefel

Sibille Everhard

Claire Dumas Bonnetain

Muriel Dahan

Florence Joly

Nourredine Ait Rahmoune

Djihane Lecheheb

Maria-Alice Borinelli-Franzoi

Emma Gillanders

Sandra Birbilaite

Gertruda Klimaviciute-Dvilevic

Danute Buteniene

Dina Janaviciene

Rita Steponaviciene

Valdas Peceliunas

Kostas Marias

Costas Papaloukas

Marion L'Hôte

Richard Price

Agnese Konusevska

Otilia Colceriu

Elisabetta Zanon

Alexandra Eni

Mareike Rutenkröger

Rosangela Caruso

Martino Belvederi Murri

Clemens Farkas

Jolana Wager-Skacel

Stefanie Hechenberger

Maria Die Trill

Lotte van der Weijst

Nikolina Dodlek

Andela Skarpa

Chiara De Tomasso

Nigel Olisa

Erik Van der Eycken

Cecilia Angulo

Elysie Nguyen

Carina Schneider

Barbara Brunmair

Anica Vrdoljak

Tihana Govorčinović

Ira Renko

Katarina Lisjak

Evgenia Aleksandrova

Anna Lyubenova

Aleiksander Milanov

Olexiy Kovalyov

Natalya Guba

Natalya Mosol

Sergey Kozhukhov

Dominika Vrbnjak

Majda Pajnkihar

Gregor Stiglic

Kasandra Musovič

Alessandro Laviano

Diana Garcia-Ramon

Sarah Collen

Elena Plana

Adrian Brîndușan

Roxana Plesoianu
